# Supplementary material for: The protamines of the spider Steatoda sp. provide an example of liquid–liquid phase separation chromatin transitions during spermiogenesis
Source: Development. 2024 Nov 18;151(22):dev203134. doi: 10.1242/dev.203134 (PMC11607694; doi:10.1242/dev.203134)
Supplement: Supplementary information [file develop-151-203134-s1.pdf]

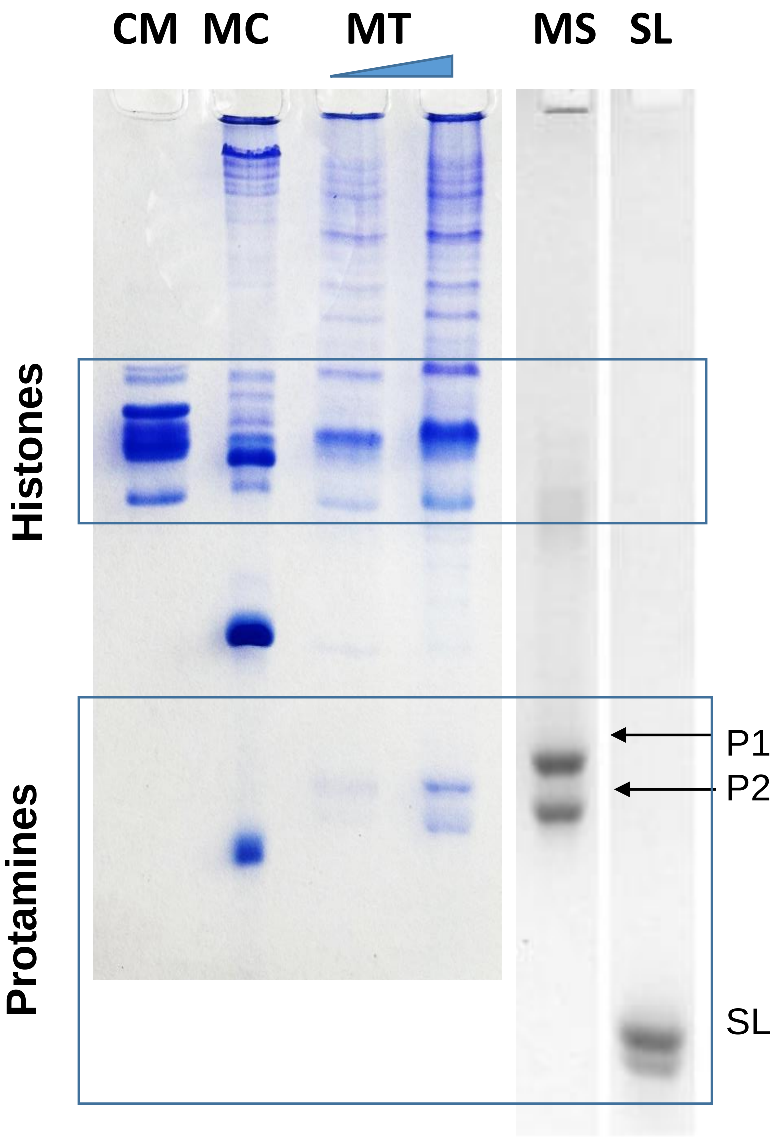

Fig. S1. Extraction of protaminesfrom 20 milligrams of mouse tests using the method described in this paper. AU- PAGE analysis showing the mouse testis (MT) analysis of the protaminesextracted by this method (one single and double loading are shown) in comparison to the proteins extracted by conventional HClextraction from: CM, chicken erythrocyte used as a histone marker, MC, *M. californianus* male gonad. The image of the gel is shown in comparison to the SNBPs extracted from mature mouse sperm (MS) obtained by a conventional method used also extract cysteine-containing protaminesstarting from larger amounts of material (Ausio et al., 2014). SL, salmine protamine as in Fig. 1.

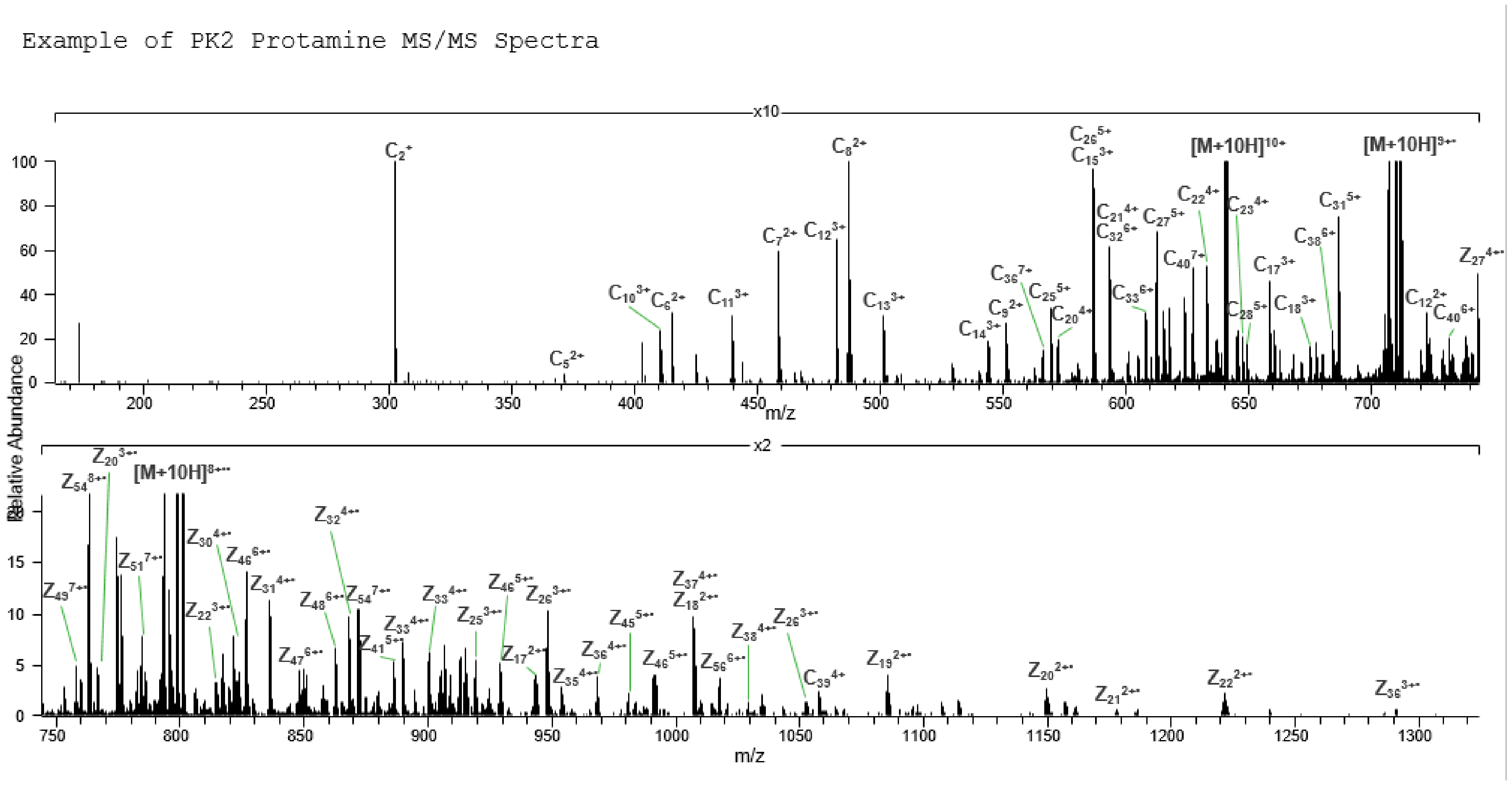

Fig. S2. Example of PK2 Protamine MS/MS Spectra. Precursor ions at 640.89 *m/z* (z=10) were fragmented by ETD to produce the MS^2 and selected abundant fragment ions are labeled.

+7 c ions	+6 c ions	+5 c ions	+4 c ions	+3 c ions	+2 c ions	+1 c ions		Sequence		+1 z ions	+2 z ions	+3 z ions	+4 z ions	+5 z ions	+6 z ions	+7 z ions
						146.1288	1	K	58	6395.8019	3198.4046	2132.6055	1599.7059	1279.9662	1066.8064	914.5494
						302.2299	2	R	57	6251.6882	3126.3477	2084.5676	1563.6775	1251.1435	1042.7874	893.9617
					229.6691	458.3310	3	R	56	6095.5871	3048.2972	2032.5339	1524.6522	1219.9232	1016.7706	871.6615
					307.7197	614.4321	4	R	55	5939.4860	2970.2466	1980.5002	1485.6270	1188.7030	990.7537	849.3614
					371.7672	742.5271	5	K	54	5783.3849	2892.1961	1928.4665	1446.6017	1157.4828	964.7369	827.0612
					415.2832	829.5591	6	S	53	5655.2899	2828.1486	1885.7682	1414.5779	1131.8638	943.3877	808.7619
					458.7992	916.5911	7	S	52	5568.2579	2784.6326	1856.7575	1392.8199	1114.4574	928.8824	796.3288
					487.3099	973.6126	8	G	51	5481.2259	2741.1166	1827.7468	1371.0619	1097.0510	914.3770	783.8956
				367.9074	551.3574	1101.7076	9	K	50	5424.2044	2712.6058	1808.7397	1356.8066	1085.6467	904.8735	775.7497
				410.6057	615.4049	1229.8025	10	K	49	5296.1094	2648.5584	1766.0413	1324.7828	1060.0277	883.5243	757.4504
				439.6164	658.9209	1316.8346	11	S	48	5168.0145	2584.5109	1723.3430	1292.7591	1034.4087	862.1751	739.1512
				482.3147	722.9684	1444.9295	12	K	47	5080.9824	2540.9949	1694.3323	1271.0011	1017.0023	847.6698	
				501.3218	751.4791	1501.9510	13	G	46	4952.8875	2476.9474	1651.6340	1238.9773	991.3833	826.3206	
				544.0202	815.5266	1630.0460	14	K	45	4895.8660	2448.4366	1632.6269	1224.7220	979.9790	816.8171	
				586.7185	879.5741	1758.1409	15	K	44	4767.7711	2384.3892	1589.9285	1192.6982	954.3600	795.4679	
				615.7292	923.0901	1845.1729	16	S	43	4639.6761	2320.3417	1547.2302	1160.6745	928.7410	774.1187	
				658.4275	987.1376	1973.2679	17	K	42	4552.6441	2276.8257	1518.2195	1138.9165	911.3346	759.6134	
				677.4346	1015.6483	2030.2894	18	G	41	4424.5491	2212.7782	1475.5212	1106.8927	885.7156	738.2642	
			540.3515	720.1330	1079.6958	2158.3843	19	K	40	4367.5276	2184.2675	1456.5141	1092.6374	874.3113	728.7607	
			572.3753	762.8313	1143.7433	2286.4793	20	K	39	4239.4327	2120.2200	1413.8157	1060.6136	848.6924	707.4115	
			594.1333	791.8420	1187.2593	2373.5113	21	S	38	4111.3377	2056.1725	1371.1174	1028.5899	823.0734	686.0623	
			633.1586	843.8757	1265.3099	2529.6124	22	R	37	4024.3057	2012.6565	1342.1067	1006.8319	805.6670	671.5570	
			647.4139	862.8828	1293.8206	2586.6339	23	G	36	3868.2046	1934.6059	1290.0730	967.8066	774.4467	645.5402	
		543.7516	679.4377	905.5811	1357.8681	2714.7289	24	K	35	3811.1831	1906.0952	1271.0659	953.5512	763.0424	636.0366	
		569.3706	711.4614	948.2795	1421.9156	2842.8238	25	K	34	3683.0881	1842.0477	1228.3676	921.5275	737.4234	614.6874	
		586.7770	733.2194	977.2901	1465.4316	2929.8559	26	S	33	3554.9932	1778.0002	1185.6692	889.5038	711.8045	593.3383	
		612.3960	765.2432	1019.9885	1529.4790	3057.9508	27	K	32	3467.9611	1734.4842	1156.6586	867.7457	694.3981		
		623.8003	779.4985	1038.9956	1557.9898	3114.9723	28	G	31	3339.8662	1670.4367	1113.9602	835.7220	668.7791		
	541.3506	649.4193	811.5223	1081.6939	1622.0373	3243.0672	29	K	30	3282.8447	1641.9260	1094.9531	821.4666			
	562.6998	675.0383	843.5460	1124.3923	1686.0847	3371.1622	30	K	29	3154.7498	1577.8785	1052.2548	789.4429			
490.6039	572.2033	686.4426	857.8014	1143.3994	1714.5955	3428.1837	31	G	28	3026.6548	1513.8310	1009.5564	757.4192			
508.9032	593.5525	712.0615	889.8251	1186.0977	1778.6430	3556.2786	32	K	27	2969.6333	1485.3203	990.5493	743.1638			
521.3363	608.0578	729.4680	911.5831	1215.1084	1822.1590	3643.3107	33	S	26	2841.5384	1421.2728	947.8510	711.1400			
529.4823	617.5614	740.8722	925.8385	1234.1156	1850.6697	3700.3321	34	G	25	2754.5063	1377.7568	918.8403				
547.7815	638.9106	766.4912	957.8622	1276.8139	1914.7172	3828.4271	35	K	24	2697.4849	1349.2461	899.8331				
566.0808	660.2597	792.1102	989.8860	1319.5122	1978.7647	3956.5221	36	K	23	2569.3899	1285.1986	857.1348				
578.5140	674.7651	809.5166	1011.6440	1348.5229	2022.2807	4043.5541	37	S	22	2441.2949	1221.1511	814.4365				
586.6599	684.2687	820.9209	1025.8993	1367.5300	2050.7914	4100.5755	38	G	21	2354.2629	1177.6351	785.4258				
604.9592	705.6178	846.5399	1057.9231	1410.2284	2114.8389	4228.6705	39	K	20	2297.2415	1149.1244	766.4187				
627.2593	731.6347	877.7601	1096.9484	1462.2621	2192.8894	4384.7716	40	R	19	2169.1465	1085.0769	723.7203				
645.5586	752.9838	903.3791	1128.9721	1504.9604	2256.9369	4512.8666	41	K	18	2013.0454	1007.0263	671.6866				
667.8588	779.0007	934.5994	1167.9974	1556.9941	2334.9875	4668.9677	42	R	17	1884.9504	942.9788					
680.2919	793.5060	952.0058	1189.7554	1586.0048	2378.5035	4755.9997	43	S	16	1728.8493	864.9283					
702.5921	819.5229	983.2260	1228.7807	1638.0385	2456.5541	4912.1008	44	R	15	1641.8173						
720.5921	840.8720	1008.8450	1260.8044	1680.7368	2520.6015	5040.1958	45	K	14							
							46-57	_	13-2							
914.5494	1066.806	1279.9662	1599.7059	2132.606	3198.405	6395.802	58	X	1							

Fig. S3. The precursor ions at 640.89 *m/z* (z=10) were fragmented by ETD and identified fragment masses are highlighted in pink.

Development • Supplementary information

Table S1. Table of Proteins found in various samples from *S. nobilis* using LC-MS/MS analysis.

Table of Proteins found in various samples from *S. nobilis* using LC-MS/MS analysis.

Sample(s)	Mass+1 (Da)	Sequence Identified	Homologous Protein, Species
Unfractionated	6359	KRRRSRGGKKSKKSKGKKSKKSKKSKGKGKKSKGKKSGGRRRRRSKxxxxxxxxxxxx	histone 1 and histone 5 domains
Unfractionated	6396	KRRRKSSGKKSKGKKSKGKKSRGKKSKGKKGKSGKKSGKRKRSRKxxxxxxxxxxxxx	N/A
Unfractionated and Fraction 0	4808	acMDKKPDDKGKPGEKKPELGKPGEKKPDDKggKPGEKKPEEKGPK	Bacterial
Unfractionated and Fraction 0	4937	acMDKKPDDKGKPGEKKPELGKPGEKKPDDKggKPGEKKPEEKGPKK	Bacterial
Unfractionated and Fraction 0	5665	acMKEKPDDKGKPGEKKPEGPKPGEKKPEPGKPGEKKPGEPKPGEKKPEEKGPK	Bacterial
Unfractionated and Fraction 0	5794	acMKEKPDDKGKPGEKKPEGPKPGEKKPEPGKPGEKKPGEPKPGEKKPEEKGPKK	Bacterial
Unfractionated and Fraction 1	7862	AKSKNHTNHNQNRKDHRNGIKRPHRFRKESMKGVDPKFLKNLKFARKHNKKLRKQLKELTKEKPA	60S Ribosomal protein L29 [Stegodyphus mimosarum]
Unfractionated and Fraction 1	6705	KVHGSLARAGKVKGQTPKVEKQEKKKKKTGRAKRRMQFNRRFVNVVVTFGRKKGPNSNS	40S Ribosomal Protein S30 [Caerostris darwini]
Unfractionated and Fraction 1	9329	PQGSHMKAGKLKPGGKVVQLKSVKGSKSVTKKSVGGKLAPQRKKLqkaLESTLKAREATDRALRA GGSSATADKKVAGAAKGKAGRKGKKK	Histone H1.M6. 2 [Parasteatoda tepidariorum]
Unfractionated and Fraction 1	13643	diMePPQASGKAVKKAGKAQKAVRAGDKKKRKKRRKESFAIYIYKVLKQVHPDTGISSKAMSIMNS FVNDIFERIAAESSRLAHYNKRSTITSREIQTAVRLLLPGELAKHAVSEGTKAVTKYTSSK	Histone H2B [Trichonephila clavata]
Unfractionated and Fraction 1	9466	shGSDGDRQRG(VYKGKKDHGGDGGRKQGR)...KAKK	Ribosomal pProteins
Unfractionated and Fraction 1	6508	KRRRSRGGKKSKKSKGKKSKKSKKSKGKGKKSKGKKSGGRRRRRSKxxxxxxxxxx	Hypothetical Protein AVEN_19411-1 [Araneus ventricosus]
Fraction 5	14145	(acAS)QKSRPQRSKYLATASTmDHARHGFLxxxxxxxxxxGGDRSRgspmAARR	Myelin Basic Protein Isoform 6 [Mus musculus]
Fraction 5	17725	NPLVGHGHLVNTGVSARAQSQDFVghp	Adult-Specific Rigid Cuticular Protein 15.7-like [Parasteatoda tepidariorum]

Development • Supplementary information
