# Supplementary material for: The protamines of the spider Steatoda sp. provide an example of liquid–liquid phase separation chromatin transitions during spermiogenesis
Source: Development. 2024 Nov 18;151(22):dev203134. doi: 10.1242/dev.203134 (PMC11607694; doi:10.1242/dev.203134)
Supplement: Supplementary file 1 [file develop-151-203134_review_history.pdf]

The protamines of the spider *Steatoda* sp. provide an example of liquid-liquid phase separation chromatin transitions during spermiogenesis

Melissa R Leyden, Peter Michalik, Luciana Baruffaldi, Susheen Mahmood, Ladan Kalani, Donald F Hunt, Jose Maria Eirin-Lopez, Maydianne C.B. Andrade, Jeffrey Shabanowitz and Juan Ausió
DOI: 10.1242/dev.203134

Editor: Haruhiko Koseki

Review timeline
Original submission: 4 June 2024
Editorial decision: 1 August 2024
First revision received: 20 September 2024
Accepted: 3 October 2024

Original submission

First decision letter

MS ID#: DEVELOP/2024/203134

MS TITLE: The protamines of the noble false widow spider Steatoda nobilis provide an example of liquid-liquid phase separation chromatin transitions during spermiogenesis

AUTHORS: Melissa R Leyden, Peter Michalik, Luciana Baruffaldi, Susheen Mahmood, Ladan Kalani, Donald F Hunt, Jose Maria Eirin-Lopez, Maydianne C.B. Andrade, Jeffrey Shabanowitz, and Juan AusiÃ³

I have now received all the referees' reports on the above manuscript, and have reached a decision. The referees' comments are appended below, or you can access them online: please go to BenchPress and click on the 'Manuscripts with Decisions' queue in the Author Area.

As you will see, the referees express considerable interest in your work, but have some significant criticisms and recommend a substantial revision of your manuscript before we can consider publication. If you are able to revise the manuscript along the lines suggested, which may involve further experiments, I will be happy receive a revised version of the manuscript. Your revised paper will be re-reviewed by one or more of the original referees, and acceptance of your manuscript will depend on your addressing satisfactorily the reviewers' major concerns. Please also note that Development will normally permit only one round of major revision. If it would be helpful, you are welcome to contact us to discuss your revision in greater detail. Please send us a point-by-point response indicating your plans for addressing the referees’ comments, and we will look over this and provide further guidance.

Please attend to all of the reviewers' comments and ensure that you clearly highlight all changes made in the revised manuscript. Please avoid using 'Tracked changes' in Word files as these are lost in PDF conversion. I should be grateful if you would also provide a point-by-point response detailing how you have dealt with the points raised by the reviewers in the 'Response to Reviewers' box. If you do not agree with any of their criticisms or suggestions please explain clearly why this is so.

Reviewer 1

© 2024. Published by The Company of Biologists under the terms of the Creative Commons Attribution License (https://creativecommons.org/licenses/by/4.0/).

Advance summary and potential significance to field

Spermiogenesis and sperm chromatin composition remain largely unexplored in arthropods, and particularly in chelicerates, where almost nothing is known. In this report, Leyden et al. identified putative protamines from the spider Steatoda nobilis (Aranea) through sperm chromosomal proteins purification and analysis by Mass Spectrometry (MS) (Fig.1). They show that these protamines resemble the highly basic C-terminal region of a histone H5 candidate from a related species (Fig.2). They performed electron microscopy on spermatid and sperm nuclei of a yet different spider species, thus revealing the formation of lamellae during the compaction of sperm chromatin (Fig. 3). They finally speculate on a tentative scenario of the histone-to-protamine transition during spider spermiogenesis (Fig.4).

Comments for the author

Spermiogenesis and sperm chromatin composition remain largely unexplored in arthropods, and particularly in chelicerates, where almost nothing is known. The lead author and colleagues are highly experienced with the purification and identification of vertebrate and invertebrate SNBPs and there is little doubt that the identified proteins in Fig. 1 are indeed bona fide S. nobilis protamines. However, besides these essentially descriptive findings, the rest of the study appears highly speculative and several conclusions are drawn without any evidence (see my comments below). Another problem with this manuscript is that the literature on insect (essentially Drosophila) protamines is largely ignored by the authors. Finally, the somehow disparate use of different spider species (without justification) for MS, molecular evolution or microscopy, somehow affects the robustness of the study.

Main comments:

- FIGURE 1

Fig1C: The panel showing Apis mellifera protamines has already been published by the authors, in contrast to what is indicated in the figure legend. The SL panel is also reused but not commented. Are the MS raw data available? What is the proportion of identified proteins that are not predicted to be nuclear?

- FIGURE 2

The authors used a method to specifically extract cysteine-containing protamines. However, there are no cysteines in the identified spider protamines. The authors should comment on this apparent contradiction.

P.9: "A genome BLAST...": Is the indicated molecular weight of the A. ventricosus protein corresponds to the full protein or its C-terminus? Please clarify.

Fig. 2C: The authors identified other similar proteins in spider genomes: are these H1/H5 or protamines?

I am not convinced by the proposed alignment in Fig. 2B which seems to essentially involve stretches of basic residues (K,R). Have the authors tried to perform a structural alignment of these protamines with the H1/H5 winged-helix domain?

- FIGURE 3

This figure includes beautiful EM characterizations of spider spermatogenesis clearly showing the lamellae structure during compaction. It is not clear if sperm remain encapsulated during transfer in this species.

- FIGURE 4

© 2024. Published by The Company of Biologists under the terms of the Creative Commons Attribution License (https://creativecommons.org/licenses/by/4.0/).

The legend and conclusions are entirely speculative: there are no evidence for histone acetylation, protamine phosphorylation or protamine processing (the precursor has not been found in this study). Some of these points could be solved by alternative approaches (immunofluorescence for acetylated histones and to confirmation of full histone elimination) and the putative precursor protamine could be searched by analyzing testes, rather than pedipalps.

- The authors seem to ignore the literature on Drosophila protamines and the manuscript contains several incorrect statements that should be corrected by referencing to the following papers:
- SNBP phosphorylation: see Zhang et al, Nat Commun, 2023 (Drosophila).
- SNBP processing: see Kimura and Loppin, Open Biology, 2016.
- Role of SNBP cysteines and disulfide bonds: see Tirmarche et al., Nat Commun, 2016; Emelyanov & Fyodorov, 2016.
- Diversity of Drosophila protamines: see Chang et al., eLife, 2023
- The most striking feature of Drosophila (and other insects) SNBPs is the presence of one or two MST-HMG-box: Doyen et al., Cell Reports, 2015; Chang et al., eLife, 2023.

Additional comments:

- It is not clear if the authors distinguish sperm-specific histone-type SNBPs (i.e. testis-specific histone variants) from the simple absence of histone replacement during spermiogenesis in some species (somatic histones).

- About the hypothesis of a recurrent H1 origin of protamines, the original idea that the Drosophila PL Mst77F resemble H1 or HILS1 (Rathke et al., 2014) has later been refuted (Doyen et al., Cell Reports, 2015).

- There is no explanation regarding the choice of the species studied.

- Basic description of spider spermatogenesis and sperm transfer in spiders should be included earlier in the text for non-specialist readers.

- P7, the sentence starting with: "The former provides..." is difficult to read and probably incorrect.
- P13. "The occurrence of a protamine-like arginine..." This formulation in unclear and the species is not indicated.

- P14. The sentence "As we had earlier proposed..." , about the possible recurrent losses of protamines genes. On what ground this hypothesis is put forward, instead of the opposite possibility, i.e. the emergence of new protamine genes?

- P14. "The evolutionary causes are unclear..." but there are hypotheses: see Chang et al., eLife, 2023.

- What is the relevance of the paragraph on putative bacterial contaminating proteins identified in MS, assuming that it is already known that pedipalps only contain mature sperm?

- Discussion, please correct the first sentence.

- Sinodal decomposition should be defined earlier in the text.

- Fig 1A: The abbreviations for California mussels (MC) and chicken erythrocytes (CM) are confusing.

- Fig. 2C: Replace the incorrect capital letters in some of the species names

Reviewer 2

Advance summary and potential significance to field

© 2024. Published by The Company of Biologists under the terms of the Creative Commons Attribution License (https://creativecommons.org/licenses/by/4.0/).

Despite the fact that the phylum Arthropoda represents about 80% of all animals information on their sperm nuclear basic proteins (SNBPs) is extremely limited. In this context, the paper by Leyden et al makes a significant contribution, not only by expanding our knowledge on the SNBPs in this important group but also by providing further experimental data that support the evolutionary origin of protamines from an H1(H5) precursor and that provide an example of liquid-liquid phase separation during spermiogenesis.

Comments for the author

The manuscript by Leyden et al. is worth publishing in Development, although some **minor revisions need be addressed first.**

ABSTRACT

- Stating that you have developed a method that allows the extraction of Cys-containing protamines, may lead readers to assume that the protamines from this spider contain Cys, which is not the case. I suggest rephrasing this to indicate that the method allows for the extraction of both Cys-containing and non-Cys-containing protamines.

MATERIALS AND METHODS
- Biological material: Please include the use of *S. grossa*.
- The "sperm collection" section needs to be renamed to "sperm collection for protein extraction" as the samples used for electron microscopy were collected differently.
- Pyridylethylaton method: It is unclear if you have "optimized" the method or simply "adapted" your previous method for small volumes. At least, it is not clear to me what the assays with mouse testes have optimized. Additionally, I do not understand why "insect testes" are mentioned in this section. Furhtermore specify the incubation time after adding vinyl pyridine. Is it still 90 minutes as before?.

RESULTS
- Figure 2C: Please indicate the family (Araneidae or Theriniidae) to which each shown species belongs.
- LLPS section: It is unfortunate that this part had to be conducted with another species, although I understand the difficulty of obtaining this type of biological material. However, I recommend removing "S. nobilis" from the tittle of this section to avoid misunderstandings. In the same sense, I suggest to modify the caption for figure 4 to "Model for chromatin transitions...".
On the other hand, I think it would be worth giving a brief explanation of what is meant by spinodal decomposition and nucleation chromatin condensation.
Also, please change "(Fig. 2 a-b)", "(Fig. 2c-d)" and "(Fig. 3-4)" to "(Fig. 3 2a-b)", "(Fig. 3, 2c-d)" and "(Fig. 3, 3-4)", respectively.

DISCUSSION
- SNBP distribution in Arthropoda section: In the first sentence, when you state
"The occurrence of a protamine-like arginine", do you mean "The occurrence of a protamine-like SNBP in spiders"?

Other minor comments:

- In the Materials & Methods section ("sperm collection", "protein extraction" and "pyridylethylation" sections), please correct all the "squares" appearing instead of some special symbols.
- Also, in the "Mass Spectrometry" section, please add the missing space between the numerical values and the units (e.g. 7 cm instead of 7cm).
- A parenthesis is missing at the end of the Biological Material section.
- In the second line of the Pyridylethylation section, the units of concentration for KCl are missing (it currently says just 60 KCl). Also, change
"min." to "min"
- p. 12: a word is missing in the first sentence of the Discussion section ("An early XXX of SNBPs")
- p. 13: "corelate" should be "correlate"
- p. 23: "S. nobilis" must be written in italics.

© 2024. Published by The Company of Biologists under the terms of the Creative Commons Attribution License (https://creativecommons.org/licenses/by/4.0/).

Reviewer 3

Advance summary and potential significance to field

I find a very interesting work not only for the new method of extraction but also because the work contributes to filling the gap relative to SNBP in Arthropoda.

Comments for the author

In the article titled "The protamines of the noble false widow spider Steatoda nobilis provide an example of liquid-liquid phase separation chromatin transitions during spermiogenesis " the authors analyze the SNBP of the noble false widow spider Steatoda nobilis (Order Araneae, Family Theridiidae) and develope a protein extraction method that allows the extraction of cysteine-containing protamines suitable for the preparation and analysis of SNBPs from samples where the amount of starting tissue material is limited.

I find a very interesting work not only for the new method of extraction but also because the work contributes to filling the gap relative to SNBP in Arthropoda.
I am in favour of publication after a minor revision

The suggestions I would make are as follows:

- I suggest the authors add that these protamines bind DNA cooperatively due to the high presence of arginines as well as histone H1. I would read and cite this article: 10.1089/dna.2009.0993
- In the sperm chromatin of this organism, apart from protamine, what other proteins are there? What are the lower mobility bands in Figure 1?
- Is the electrophoretic pattern always the same and do the proteins have the same properties at all times of the year, or do they change as with Mytilus galloprovincialis according to seasonality? 10.1002/mrd.23240
- Better define the limitations of this study

First revision

Author response to reviewers' comments

Dear Dr. Koseki,
Please find attached a revised version of our manuscript: "The protamines of the noble false widow spider Steatoda nobilis provide an example of liquid-liquid phase separation chromatin transitions during spermiogenesis". In it, we have addressed the reviewers' comments in the following way (reviewers' comments are in black and our reply is in blue). In addition to a blank copy of the revised manuscript, we are including a copy where all the areas of the text that have been modified are highlighted in yellow.
We would like to thank the reviewers for their positive and encouraging comments and thoughtful suggestions.
However, before I proceed with the replies to the reviewers' comments, I would like to make a generic comment that might be useful in framing my answers below.
The main purpose of this work was to determine the sperm nuclear basic protein (SNBP) composition of spiders, an animal group that had evaded our analysis in the past but which coincidentally became available to my group several months before I was about to retire on July 1st, 2024. The implication is that this work is my last experimental research ; hence, unfortunately no more experimental work can be added as my lab is permanently closed.
As it mainly pertains to the first reviewer, It is very hard to compare this work to related work in Drosophila for many reasons. First of all, Drosophila is an insect, not an arachnid. Even within

© 2024. Published by The Company of Biologists under the terms of the Creative Commons Attribution License (https://creativecommons.org/licenses/by/4.0/).

insects, the Drosophila protamine has a very unique amino acid sequence that completely departs from the conventional arginine-rich sequences, which is characteristic of most plants and animals including other insects (Leyden et al., 2024). In that paper, we have biochemically purified and characterized the largest number of insect protamines to date (Leyden et al., 2024). All of them exhibited a canonical arginine-rich protamine composition with arginine-clusters. This is in striking contrast to Drosophila which does not contain numerous arginine residues in its protamines.
Second, fruit flies can be grown in large quantities; thousands of individuals can be grown in a short span of time, and its genetics are very well established. Neither of which is the case in arachnids. Hence, biochemically purifying the protamines from A. nobilis is not trivial, as acknowledged by reviewer 2, by stating “the difficulty of obtaining this type of biological material”. Therefore, in comparing this work to Drosophila one needs to bear all these caveats in mind.
Lastly, my lab over the years, and my extensive career in the SNBP field has demonstrated that in many animals and plants (D'Ippolito et al., 2019) protamines and protamine-like proteins are often derived from an ancestral histone H5 (a replication independent histone H1 which is present in terminally differentiated cells). This is no longer a hypothesis but a very well experimentally documented fact. See (Eirin-Lopez and Ausio, 2009) from the list of references below, for a comprehensive review.
Answers to reviewers:
Reviewer 1:
Spermiogenesis and sperm chromatin composition remain largely unexplored in arthropods, and particularly in chelicerates, where almost nothing is known. The lead author and colleagues are highly experienced with the purification and identification of vertebrate and invertebrate SNBPs and there is little doubt that the identified proteins in Fig. 1 are indeed bona fide S. nobilis protamines. However, besides these essentially descriptive findings, the rest of the study appears highly speculative and several conclusions are drawn without any evidence (see my comments below). Another problem with this manuscript is that the literature on insect (essentially Drosophila) protamines is largely ignored by the authors. Finally, the somehow disparate use of different spider species (without justification) for MS, molecular evolution or microscopy, somehow affects the robustness of the study.

See our answers below:
Main comments:

Comment 1)
- FIGURE 1

Fig1C: The panel showing Apis mellifera protamines has already been published by the authors, in contrast to what is indicated in the figure legend. The SL panel is also reused but not commented.
The source of the panel showing A. mellifera from our recently published paper on insect protamines has been added to the figure legend. SL corresponds to the protamine from the salmon (salmine) and my lab has consistently used this over the years as a protamine marker for relative gel electrophoretic mobility of SNBPS in acetic acid urea PAGE.

Comment 2) Are the MS raw data available? What is the proportion of identified proteins that are not predicted to be nuclear?
As indicated in the Materials and Methods section, the SNBPs of S. nobilis were HCl extracted and fractionated by HPLC. Individual HPLC fractions were analyzed by MS. This is different from retrieving the protamines from the whole proteome from total sperm. Hence, it is difficult to know how many proteins obtained by our analysis “are predicted not to be nuclear”. Nevertheless, all the MS data performed in this paper are available from the Donald Hunt’s lab at the University of Virginia and a table S1 is now included in the Supplementary materials showing the sequences of all the proteins identified as well as a Figure S3 showing what the raw data looks like.
Comment 3)

- FIGURE 2

The authors used a method to specifically extract cysteine-containing protamines. However, there are no cysteines in the identified spider protamines. The authors should comment on this apparent contradiction.

© 2024. Published by The Company of Biologists under the terms of the Creative Commons Attribution License (https://creativecommons.org/licenses/by/4.0/).

We have modified the first paragraph of the results section (see page 3, line 6) to make it more clear, and following the suggestion by reviewer 2, we have indicated that the current method “allows for the extraction of both Cys-containing and non-Cys-containing protamines” which is extremely beneficial when limited access of material is possible. The present re-writing should now clarify the apparent contradiction.
Comment 4)

P.9: "A genome BLAST...": Is the indicated molecular weight of the A. ventricosus protein corresponds to the full protein or its C-terminus? Please clarify.
Yes, and this has been indicated in the legend of Fig. 2B-1.

Comment 5)
Fig. 2C: The authors identified other similar proteins in spider genomes: are these H1/H5 or protamines?
They are all histone H5-like.
Comment 6)

I am not convinced by the proposed alignment in Fig. 2B which seems to essentially involve stretches of basic residues (K,R). Have the authors tried to perform a structural alignment of these protamines with the H1/H5 winged-helix domain?
We did not annotate the A. ventricosus genome; hence, we did not identify the protein as a histone H5-like but whoever annotated that sequence, did label it this way. However, given the structural concerns raised by the reviewer, we have modified Fig. 2 to introduce a new panel (Fig. 2B-2). In it we identify the amino acid sequence regions corresponding to the WHD of histone H5 (WHD1) and A. ventricosius (WHD2) in Fig. 1B-1, and using them, we determined the tertiary structure of WHD corresponding to the two sequences determined by Neurosnap AlphaFold 2. This, unequivocally shows that, indeed the annotated sequence for A. venticosus as a H5-like protein contains a WHD similar to that of chicken histone H5. Moreover, we have addressed this in the text of the revised version, see page 13 lines 3 to 5 and Figure legend to Fig. 2.
Comment 7)

- FIGURE 3

This figure includes beautiful EM characterizations of spider spermatogenesis clearly showing the lamellae structure during compaction. It is not clear if sperm remain encapsulated during transfer in this species.
Yes, the sperm remains encapsulated during the transfer from the pedipalps of the male to the female during copulation.
Comment 8)

- FIGURE 4

The legend and conclusions are entirely speculative: there are no evidence for histone acetylation, protamine phosphorylation or protamine processing (the precursor has not been found in this study). Some of these points could be solved by alternative approaches (immunofluorescence for acetylated histones and to confirmation of full histone elimination) and the putative precursor protamine could be searched by analyzing testes, rather than pedipalps.
As mentioned above, my lab is completely closed and there are no further possibilities for performing any of the experiments suggested by this reviewer. Sperm from the pedipalps was chosen in this analysis because they consist of the mature sperm before fertilization, whereas the testes do not consist of mature sperm.
Figure 4B is only used to illustrate the complexity of spinodal decomposition, and even if my lab was still open, it would require a humongous effort, which is beyond the scope of this paper. However, to indicate this clearly, we have added two sentences to the discussion section to this effect: “This is a dynamic process that, in the species where this has been observed so far, at the biochemical level” (see page 15, line 6) and “Hence, Fig 4B is not based on any experimental work carried out in this paper but on previously published paper reviewed in (Kasinsky et al., 2012)” (see page 15, lines 13-15)
Comment 9)

© 2024. Published by The Company of Biologists under the terms of the Creative Commons Attribution License (https://creativecommons.org/licenses/by/4.0/).

- The authors seem to ignore the literature on Drosophila protamines and the manuscript contains several incorrect statements that should be corrected by referencing to the following papers:
- SNBP phosphorylation: see Zhang et al, Nat Commun, 2023 (Drosophila).
- SNBP processing: see Kimura and Loppin, Open Biology, 2016.
- Role of SNBP cysteines and disulfide bonds: see Tirmarche et al., Nat Commun, 2016; Emelyanov & Fyodorov, 2016.
- Diversity of Drosophila protamines: see Chang et al., eLife, 2023
- The most striking feature of Drosophila (and other insects) SNBPs is the presence of one or two MST-HMG-box: Doyen et al., Cell Reports, 2015; Chang et al., eLife, 2023.

While we appreciate the general importance of these papers to the protamine field, as I mentioned in introductory comments preceding answers to the reviewers, this is not a Drosophila paper, let alone an insect paper. We have published our results on insect protamines in a recent paper (Leyden et al., 2024). Accordingly, in the present spider paper, Drosophila is mentioned only 2 times and it is hard to see how by additionally referencing the 6 references on Drosophila, suggested by the reviewer, will correct the incorrect statements: " several incorrect statements that should be corrected by referencing to the following papers". It is not specified which ones are the incorrect statements nor what the reviewer's statement itself means.
None of the reviewer's suggested papers have any relevance to the content of this paper nor to the two instances where the word Drosophila appears in the manuscript on page 5 line 2 from the bottom and page 8 line 8 from the bottom.
Additional comments:
Comment 10)

- It is not clear if the authors distinguish sperm-specific histone-type SNBPs (i.e. testis-specific histone variants) from the simple absence of histone replacement during spermiogenesis in some species (somatic histones).

The introduction section has now addressed this (see page 4, lines 10-13 from the top).

- About the hypothesis of a recurrent H1 origin of protamines, the original idea that the Drosophila PL Mst77F resemble H1 or HILS1 (Rathke et al., 2014) has later been refuted (Doyen et al., Cell Reports, 2015).

First of all, the histone H1/H5 origin of several protamines in non-Drosophila organisms is not a hypothesis. Research in my lab over the last 40 years has experimentally demonstrated this to be the case in many organisms such as plants, vertebrates and invertebrate organisms, including the one in this paper. As already mentioned over these pages, Drosophla protamine, unlike that of several other insects (Leyden et al., 2024) is quite unique. While our paper on insects did not provide evidence for any insect protamines analyzed by us to be related to an ancestral histone H1, neither was it related to any HMG precursor.

Comment 10)
- There is no explanation regarding the choice of the species studied.

The species were chosen on availability. Unlike Drosophila, Steatoda sp. is not a model system where large amounts of organisms are readily available.
Comment 11)

- Basic description of spider spermatogenesis and sperm transfer in spiders should be included earlier in the text for non-specialist readers.

The description already exists.

Comment 12)
- P7, the sentence starting with: "The former provides..." is difficult to read and probably incorrect.

The term "former" has been removed from the sentence and replaced by MC and SL for clarity and, as it is stated in that sentence, the MC and SL protein markers are used as a "relative estimate of Mr" which is not incorrect.(see page 7, line 7 from the bottom of the page).
Comment 13)

- P13. "The occurrence of a protamine-like arginine..." This formulation in unclear and the species is not indicated.

© 2024. Published by The Company of Biologists under the terms of the Creative Commons Attribution License (https://creativecommons.org/licenses/by/4.0/).

The sentence has been fixed to indicate that: “The occurrence of an arginine-containing protamine-like SNBP in lycosid wolf spiders was described” (see page 13 line 8 from the bottom of the page). Unfortunately, the specific name of the spider species used in the work by Rasch and Connelly is not provided in that paper.
Comment 14)

- P14. The sentence "As we had earlier proposed..." , about the possible recurrent losses of protamines genes. On what ground this hypothesis is put forward, instead of the opposite possibility, i.e. the emergence of new protamine genes?
The recurrent loss of protamine genes is better visualized in the evolution of SNBPs in fish. When looking at the evolution of these proteins within this group, which contains examples of the three protein types: Histone, Protamine-like and protamines, it is clear that in certain orders and families, the protamine genes have been lost. In this regard, we would like to encourage the reviewer to read carefully our papers on this topic. (Saperas et al., 1994; Saperas et al., 1993).
Comment 15)

- P14. "The evolutionary causes are unclear..." but there are hypotheses: see Chang et al., eLife, 2023.
As I have repeatedly mentioned earlier, this is not a Drosophila paper, and whether “Expansion and loss of sperm nuclear basic protein genes in Drosophila correspond with genetic conflicts between sex chromosomes” mechanism as proposed in (Chang et al., eLife, 2023) by Malik’s group, can be extended to other organisms including its generalization to all groups of insects, remains to be tested.
Comment 16)

- What is the relevance of the paragraph on putative bacterial contaminating proteins identified in MS, assuming that it is already known that pedipalps only contain mature sperm?
The relevance is that we were able to gather some sequence information on these proteins, for which there was no hitherto information. The reviewer’s assumption is correct.
Comment 17)

- Discussion, please correct the first sentence
The sentence has now been corrected.
Comment 18)

- Sinodal decomposition should be defined earlier in the text.
This was also suggested by reviewer 2 (see reviewer 2, Comment 7 below). Accordingly, a brief explanation of spinodal decomposition and nucleation has now been provided in page 11 lines 1-13.
Comment 19)

- Fig 1A: The abbreviations for California mussels (MC) and chicken erythrocytes (CM) are confusing.
We have been using these abbreviations in more than 80 papers published by my lab on the protamine topic and never had any issue raised in this regard.
Comment 20)

- Fig. 2C: Replace the incorrect capital letters in some of the species names
This has been taken care of.

Reviewer 2:
Comments for the author
The manuscript by Leyden et al. is worth publishing in Development, although some minor revisions need be addressed first.

Comment 1)
ABSTRACT
- Stating that you have developed a method that allows the extraction of Cys-containing protamines, may lead readers to assume that the protamines from this spider contain Cys, which is not the case. I suggest rephrasing this to indicate that the method allows for the extraction of both Cys-containing and non-Cys-containing protamines.

© 2024. Published by The Company of Biologists under the terms of the Creative Commons Attribution License (https://creativecommons.org/licenses/by/4.0/).

We have rephrased the abstract as suggested. (see, page 3 line 6)
Comment 2)
MATERIALS AND METHODS
- Biological material: Please include the use of S. grossa.
S. grossa has been included in the Biological Material section. (see page 18, lines 18-19).
Comment 3)

- The “sperm collection” section needs to be renamed to “sperm collection for protein extraction” as the samples used for electron microscopy were collected differently.
This section has been renamed (see page 18, line 3 from the bottom of the page).
Comment 4)

- Pyridylethylaton method: It is unclear if you have “optimized” the method or simply “adapted” your previous method for small volumes. At least, it is not clear to me what the assays with mouse testes have optimized. Additionally, I do not understand why “insect testes” are mentioned in this section. Furhtermore, specify the incubation time after adding vinyl pyridine. Is it still 90 minutes as before?.
All of this has now been addressed in page 20, lines 2-8 from the bottom.
Comment 5)

RESULTS
- Figure 2C: Please indicate the family (Araneidae or Theriniidae) to which each shown species belongs.
All the species shown in Fig 2C belong to the family Araneidae. This has now been clearly indicated in the legend to figure 2C.
Comment 6)

- LLPS section: It is unfortunate that this part had to be conducted with another species, although I understand the difficulty of obtaining this type of biological material. However, I recommend removing “S. nobilis” from the tittle of this section to avoid misunderstandings. In the same sense, I suggest to modify the caption for figure 4 to “Model for chromatin transitions...”.
The title of the section has been modified accordingly.
Comment 7)

On the other hand, I think it would be worth giving a brief explanation of what is meant by spinodal decomposition and nucleation chromatin condensation.
A brief explanation of spinodal decomposition and nucleation has now been provided in page 11 lines 1-13.

Also, please change “(Fig. 2 a-b)”, “(Fig. 2c-d)” and “(Fig. 3-4)” to “(Fig. 3, 2a-b)”, “(Fig. 3, 2c-d)” and “(Fig. 3, 3-4)”, respectively.
The nomenclature of these figures has been changed accordingly.

Comment 8)
DISCUSSION
- SNBP distribution in Arthropoda section: In the first sentence, when you state “The occurrence of a protamine-like arginine”, do you mean “The occurrence of a protamine-like SNBP in spiders”?
This has also been changed for clarity (see page 14, line 10 from the bottom of the page).

Comment 9)

Other minor comments:
- In the Materials & Methods section (“sperm collection”, “protein extraction” and “pyridylethylation” sections), please correct all the “squares” appearing instead of some special symbols.
In the original paper of the manuscript I am using for this correction there are no squares in any of these sections, which I am assuming correspond to the abbreviation for microliter. We will make sure that in our resubmission this error does not occur again.

© 2024. Published by The Company of Biologists under the terms of the Creative Commons Attribution License (https://creativecommons.org/licenses/by/4.0/).

- Also, in the “Mass Spectrometry” section, please add the missing space between the numerical values and the units (e.g. 7 cm instead of 7cm).
We have corrected this in our revised version.

- A parenthesis is missing at the end of the Biological Material section.
- In the second line of the Pyridylethylation section, the units of concentration for KCl are missing (it currently says just 60 KCl). Also, change “min.” to “min”
- p. 12: a word is missing in the first sentence of the Discussion section (“An early XXX of SNBPs”)
- p. 13: “corelate” should be “correlate”
- p. 23: “S. nobilis” must be written in italics.
All the above typos have been taken care of.

Reviewer 3:

***** Reviewer 3 Advance summary and potential significance to field
I find a very interesting work not only for the new method of extraction but also because the work contributes to filling the gap relative to SNBP in Arthropoda.

Reviewer 3 Comments for the author
In the article titled “The protamines of the noble false widow spider Steatoda nobilis provide an example of liquid-liquid phase separation chromatin transitions during spermiogenesis ” the authors analyze the SNBP of the noble false widow spider Steatoda nobilis (Order Araneae, Family Theridiidae) and develope a protein extraction method that allows the extraction of cysteine-containing protamines suitable for the preparation and analysis of SNBPs from samples where the amount of starting tissue material is limited.

I find a very interesting work not only for the new method of extraction but also because the work contributes to filling the gap relative to SNBP in Arthropoda.
I am in favour of publication after a minor revision

The suggestions I would make are as follows:

Comment 1)
•I suggest the authors add that these protamines bind DNA cooperatively due to the high presence of arginines as well as histone H1. I would read and cite this article: 10.1089/dna.2009.0993
We have mentioned this article on page 13, last two lines
Comment 2)

•In the sperm chromatin of this organism, apart from protamine, what other proteins are there? What are the lower mobility bands in Figure 1?
The proteins identified by MS, in addition to protamines, are now shown on the newly added supplementary Fig.2.
The lower bands see in Fifg.1 correspond to some of the low molecular weight bacterial peptides shown in this supplementary figure.
Comment 3)
•Is the electrophoretic pattern always the same and do the proteins have the same properties at all times of the year, or do they change as with Mytilus galloprovincialis according to seasonality? 10.1002/mrd.23240
In contrast to Mytilus which has two major spawning cycles in the spring and in the fall, the spider mating season occurs mainly in the fall from September to November. Unfortunately, due to the limitations in the amount of material available we were not able carry a seasonal variation study in this instance. However, we suspect that should this have been possible, we might have been able to identify a decrease in the histone H5 precursor and an increase in the two main protamine bands during season maturation. See also our answer to reviewer 1 comment 8.
Comment 4)

•Better define the limitations of this study
To this end we have added a new section at the end of the discussion which highlights the limitations and future perspectives of our work (end of page 17, beginning of page 18).

© 2024. Published by The Company of Biologists under the terms of the Creative Commons Attribution License (https://creativecommons.org/licenses/by/4.0/).

In closing, we would like to thank, one more time, the reviewers for the time they spent working on our manuscript. We hope this revised version is now suitable for acceptance for publication. Sincerely,

Juan Ausió, Professor on behalf of all the authors.

REFERENCES pertinent to the preceding rebuttal document.

D'Ippolito, R.A., Minamino, N., Rivera-Casas, C., Cheema, M.S., Bai, D.L., Kasinsky, H.E., Shabanowitz, J., Eirin-Lopez, J.M., Ueda, T., Hunt, D.F., Ausio, J., 2019. Protamines from liverwort are produced by post-translational cleavage and C-terminal di-aminopropanelation of several male germ-specific H1 histones. J Biol Chem 294, 16364-16373.
Eirin-Lopez, J.M., Ausio, J., 2009. Origin and evolution of chromosomal sperm proteins. Bioessays 31, 1062-1070.
Leyden, M.R., Gowen, B., Gonzalez-Romero, R., Eirin-Lopez, J.M., Kim, B.H., Hayashi, F., McCartney, J., Zhang, P.C., Kubo-Irie, M., Shabanowitz, J., Hunt, D.F., Ferree, P., Kasinsky, H., Ausio, J., 2024. Protamines and the sperm nuclear basic proteins Pandora's Box of insects. Biochem Cell Biol.
Saperas, N., Ausió, J., Lloris, D., Chiva, M., 1994. On the evolution of protamines in bony fish: alternatives to the "retroviral horizontal transmission" hypothesis. J Mol Evol 39, 282-295.
Saperas, N., Lloris, D., Chiva, M., 1993. Sporadic appearance of histones, histone-like proteins and protamines in sperm chromatin of bony fish. Journal of Experimental Zoology 265, 575-586.

Second decision letter

MS ID#: DEVELOP/2024/203134

MS TITLE: protamines of the spider Steatoda sp. provide an example of liquid-liquid phase separation chromatin transitions during spermiogenesis

AUTHORS: Melissa R Leyden, Peter Michalik, Luciana Baruffaldi, Susheen Mahmood, Ladan Kalani, Donald F Hunt, Jose Maria Eirin-Lopez, Maydianne C.B. Andrade, Jeffrey Shabanowitz, and Juan Ausió

I am happy to tell you that your manuscript has been accepted for publication in Development, pending our standard publication integrity checks.

© 2024. Published by The Company of Biologists under the terms of the Creative Commons Attribution License (https://creativecommons.org/licenses/by/4.0/).
